# No evidence for MHC‐based mate choice in wild giant pandas

**DOI:** 10.1002/ece3.4419

**Published:** 2018-08-01

**Authors:** Lijun Yu, Yonggang Nie, Li Yan, Yibo Hu, Fuwen Wei

**Affiliations:** ^1^ CAS Key Laboratory of Animal Ecology and Conservation Biology Institute of Zoology Chinese Academy of Sciences Beijing China; ^2^ University of Chinese Academy of Sciences Beijing China; ^3^ Center for Excellence in Animal Evolution and Genetics Chinese Academy of Sciences Kunming China

**Keywords:** adaptive variation, *Ailuropoda melanoleuca*, male–male competition, sexual selection

## Abstract

Major histocompatibility complex genes (MHC), a gene cluster that controls the immune response to parasites, are regarded as an important determinant of mate choice. However, MHC‐based mate choice studies are especially rare for endangered animals. The giant panda (*Ailuropoda melanoleuca*), a flagship species, has suffered habitat loss and fragmentation. We investigated the genetic variation of three MHC class II loci, including DRB1, DQA1, and DQA2, for 19 mating‐pairs and 11 parent‐pairs of wild giant pandas based on long‐term field behavior observations and genetic samples. We tested four hypotheses of mate choice based on this MHC variation. We found no supporting evidence for the MHC‐based heterosis, genetic diversity, genetic compatibility and “good gene” hypotheses. These results suggest that giant pandas may not use MHC‐based signals to select mating partners, probably because limited mating opportunities or female‐biased natal dispersal restricts selection for MHC‐based mate choice, acknowledging the caveat of the small sample size often encountered in endangered animal studies. Our study provides insight into the mate choice mechanisms of wild giant pandas and highlights the need to increase the connectivity and facilitate dispersal among fragmented populations and habitats.

## 1 INTRODUCTION

Sexual reproduction is an advanced but relatively inefficient reproductive style. This paradox of sexual reproduction has been interpreted with several hypotheses, including the Red Queen hypothesis. This hypothesis states that new combinations of genes are required through the selection of certain partners to resist the currently dominating parasites, while parasites evolve rapidly (Bell, 1982; Van Valen, 1973). Thus, a female is predicted to select a male who possesses resistance genes that would, in combination with her own genes, provide offspring with the best immune response against the dominant parasites. The major histocompatibility complex (MHC) genes, a gene‐rich and remarkably polymorphic genomic region, play a crucial function in immune response and parasite resistance (Klein, 1986) as well as in behavioral ecology and population health in vertebrates (Sommer, 2005). Thus, the MHC genes are an important candidate genetic marker for mate choice.

Researchers have found a strong correlation between MHC genes and different physical signals. For example, Santos, Kellermann, Uchanska‐Ziegler, and Ziegler (2010) found largely conserved linkage between distinct odorant receptor genes and MHC genes among 16 vertebrate species, suggesting that the MHC genes function in a concerted fashion. Huchard, Raymond, et al. (2010) found that in a wild baboon population (*Papio ursinus*), MHC‐based sexual signals were conveyed via physical condition. This evidence shows that females can choose mates who harbor special MHC genes through physical signals. In addition, research related to postcopulatory mate choice proposed that MHC haplotypes expressed on the surface of mature sperm can be identified by females, which remains to be proved and is not our main concern here. Thus, the offspring attain additional fitness benefits in precopulatory mate choice through MHC‐mediated selective mechanisms in terms of immune resistance to disease and adaptations to the changing environment, rendering sexual selection evolutionarily favorable.

Previous research on MHC‐based mate choice has identified four main genetic mechanisms underlying precopulatory mate choice in vertebrates (Brown, 1997; Hamilton & Zuk, 1982; Kempenaers, 2007; Møller & Alatalo, 1999; Penn, Damjanovich, & Potts, 2002; Puurtinen, Ketola, & Kotiaho, 2005). First, mate choice is mediated through heterosis, which supposes that the heterozygotes of a targeted MHC locus are preferred over homozygotes. A direct benefit of heterozygosity in the MHC genes is the reduced risk of disease in offspring. Heterozygosity also provides indirect benefits by allowing the individual to be a carrier of rare alleles (Hughes & Nei, 1988). Second, mate choice occurs based on MHC genetic diversity, measured by the number of variable sites at a targeted MHC locus (Lenz, Wells, Pfeiffer, & Sommer, 2009). Third, animals prefer to mate with MHC‐dissimilar partners (i.e., genetic compatibility hypothesis), which is expected to produce offspring with higher fitness (Raveh et al., 2014; Sommer, 2005). In addition, if the choice of MHC gene is based on the immune effect, the partner's MHC allele is important. The optimizing hypothesis assumes that choosing a mate with the most appropriate number of MHC diversity or dissimilarity, but not the maximum, will benefit future generations, that is, preferred in some animals (Milinski, 2006; Wegner, Kalbe, Kurtz, Reusch, & Milinski, 2003), which is similar to the genetic compatibility hypothesis. Fourth, the “good gene” hypothesis emphasizes the choice for specific MHC alleles. For example, sires of wild and captive tuco‐tucos were shown to carry distinctive alleles (Cutrera, Fanjul, & Zenuto, 2012).

The giant panda (*Ailuropoda melanoleuca*) is one of the most threatened animals, although the IUCN recently downlisted it from “endangered” to “vulnerable” based on population and habitat recovery (Swaisgood et al., 2016). The 4th national survey of giant pandas estimated the global population size at 1,864 individuals living in 33 fragmented populations (State Forestry Administration, 2015). A recent genetic study also found that the level of inbreeding in wild giant pandas was greater than expected (Hu et al., 2017). However, multiple studies have shown that giant pandas still harbor relatively high genetic diversity and evolutionary potential (Zhang et al., 2007), suggesting that some mechanisms may maintain genetic variation in the population. Studies have reported that MHC‐based mate choice mechanisms function in shaping genetic diversity. For example, Santos, Michler, and Sommer (2017) found that the MHC‐based mechanism in mate choice may allow raccoons to overcome inbreeding constraints during population expansion.

Wild giant pandas are rare and elusive and live in complex habitats, making direct field observations extremely difficult. Knowledge of wild giant panda reproduction is inferred from three long‐term radio/GPS‐telemetry studies (Nie, Swaisgood, Zhang, Liu, & Wei, 2012; Pan et al., 2014; Schaller, Hu, Pan, & Zhu, 1985). The courting and mating behaviors of giant pandas often occur from the end of February to the end of April. At first, males gather around an estrous female in a mating site. Each mating site comprises one female and at least one male. In most cases, males determine dominance through combat, and the winner receives access to the female. The female usually mates with the male winner, but occasionally mating opportunities will be granted to other males. A previous study on captive giant pandas showed that higher copulation and birth rates can be obtained if females are allowed to mate with their preferred partners, implying that mate choice may occur in wild pandas (Martin‐Wintle et al., 2015). In addition, as a polygynous species, female giant panda lives by herself during the gestation, parturition and infant rearing period. Therefore, based on the parental investment theory (Trivers, 1972), females tend to be more active in mate choice to ensure maximum reproductive benefits.

The genetic diversities of MHC genes as adaptive genetic markers have been assessed among giant panda populations (Zeng, Yu, Pan, Wan, & Fang, 2005; Zhu, Ruan, Ge, Wan, & Fang, 2007; Zhu, Sun, et al., 2013; Zhu, Wan, Yu, Ge, & Fang, 2013). In particular, Zhang, Wu, Hu, Wu, and Wei (2015) analyzed the correlation between MHC genetic diversity and parasite infection in wild giant pandas and found a target MHC gene associated with parasite infection. However, the role of MHC genes in mate choice of giant pandas remains unexplored. In our study, we implemented a combination of long‐term field observations of reproductive behavior and noninvasive genetic sampling (2006‐2016) in the Foping and Changqing Nature Reserves to test whether MHC‐based mechanisms play a role in male–male competition and mate choice in wild giant pandas.

## 2 MATERIALS AND METHODS

### 2.1 Study area, behavior observation, and sample collection

**Figure 1:**
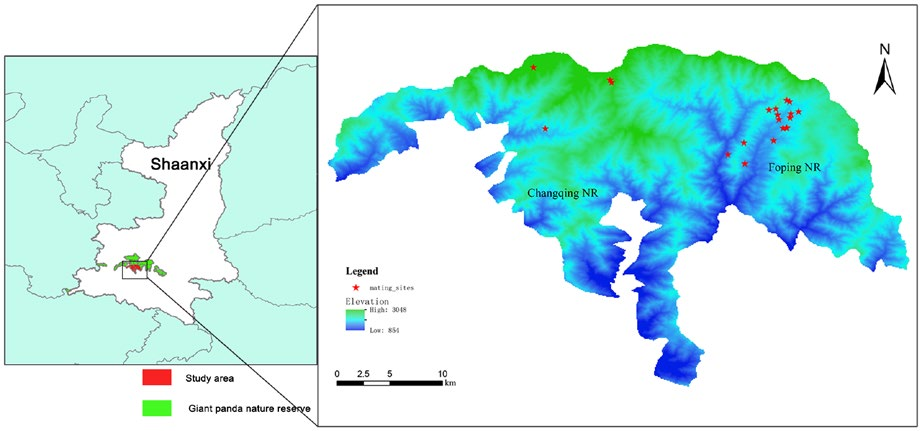
The Foping and Changqing Nature Reserves and the locations of 19 mating sites found from 2008 to 2016 [Colour figure can be viewed at http://wileyonlinelibrary.com]

We implemented this study in the Foping nature reserve (Foping) and the adjacent Changqing nature reserve (Changqing) in Shaanxi Province (Figure 1), located on the south slope of the Qinling Mountains. According to the 3rd and 4th national surveys of giant pandas (State Forestry Administration, 2006, 2015), Foping (33°33′–33°46′N, 107°40′‐107°55′E) is estimated to have 67–76 giant pandas (excluding cubs), which is approximately 19.4%–27.6% of the entire Qinling population, and Changqing (33°26′–33°43′N, 107°25′–107°45′E) has 52–57 giant pandas (excluding cubs), comprising 16.5%–18.9% of the entire Qinling population. Considering that these reserves have higher population densities of giant pandas than other nature reserves, we chose them to ensure that we found as many mating sites (i.e., the aggregating sites of estrous males and female) as possible.

Two methods, tracking GPS‐collared pandas (Wei et al., 2014) and following the unique vocalizations of courting or mating pandas, were used to search for mating sites from 2008 to 2016 in Foping and from 2008 to 2010 in Changqing (see details in Hu et al., 2017). We identified the estrous female, dominant male (i.e., the male who won the combat), and the subordinate males (i.e., other males who participated in combat) by observing behavioral and spatial position cues (Pan et al., 2014; Schaller et al., 1985). After the reproductive activity was over, we collected fresh feces and shed hair samples using sterile polyethylene gloves following previous methods (Hu, Zhan, Qi, & Wei, 2010; Zhan et al., 2006). We also collected samples of mother‐cub pairs through opportunistic encounters in the field, the tracking of collared females, and the monitoring of natal dens in winter. Feces samples of mother‐cub pairs were attributed to either mothers or cubs based on fecal size. If the cub was not old enough to defecate, we collected the cub's hair sample when conditions allowed (see details in Hu et al., 2017). It was not possible to record data blind because our study involved focal animals in the field.

### 2.2 Identification and sex determination of individuals

We extracted total genomic DNA from feces using the QIAamp DNA Stool Mini kit (QIAGEN, Hilden, Germany) and from hair using proteinase k in a PCR‐compatible buffer (Allen et al., 1998). Blank controls were performed for both extractions and downstream amplifications. We used 14 giant panda‐specific microsatellite loci to genotype the DNA samples (see details in Hu et al., 2017). To obtain reliable genotypes, we used a multi‐tube amplification approach (Taberlet et al., 1996). First, we amplified each DNA sample three times, and if the genotype could not be determined, we performed two additional amplifications. PCR volumes and conditions as well as microsatellite genotyping are detailed in Hu et al. (2017). After obtaining multi‐locus combined genotypes, we identified individuals following Zhan et al. (2006). We used MICRO‐CHECKER (Van Oosterhout, Hutchinson, Wills, & Shipley, 2004) to detect the presence of genotyping errors, such as null alleles, large allele dropouts, and stuttering. No genotyping errors were detected in our final dataset. We used a Y‐linked sexing marker (ZX1, 210‐bp) in combination with an X/Y‐linked amplification control (ZFX/ZFY, 130‐bp) to determine the sex of each sample (Hu et al., 2017).

### 2.3 Paternity assignment

For mother‐cub pairs, we performed maximum likelihood‐based paternity assignment based on the microsatellite data using Cervus v3.0.7 (Kalinowski, Taper, & Marshall, 2007). Paternity was assigned at both strict (95%) and relaxed (80%) confidence levels (see details in Hu et al., 2017).

### 2.4 MHC gene genotyping

The second exons of the three MHC class II loci (DRB1, DQA1, and DQA2) were selected for mate choice study of giant pandas because of three reasons: Firstly, MHC class II loci are the most reported loci involved in immune response, especially against extra‐cellular parasites (Sommer, 2005); secondly, based on previous studies, the second exons of the three MHC class II loci (DRB1, DQA1, and DQA2) can be correctly amplified without the risk of falsely amplifying other paralogous loci (Chen et al., 2010; Zhang et al., 2015; Zhu et al., 2007), which is very important for reliable biological analysis; and thirdly, these loci were shown to have normal expression levels (Chen et al., 2010). Exons were amplified from DNA extracted from feces or hair. At the MHC class II DRB1 locus, all samples were genotyped using the primer pairs DRB1up (5′‐AAGGGCGAGTGCTACTTCAC‐3′) and DRB1down (5′‐CCGGATGAGTCTGTCTCACA‐3′; Zhang et al., 2015). At the MHC class II DQA loci (DQA1 and DQA2), all samples were genotyped using the primer pairs DQAup (5′‐GCTGACCATGTTGCTTACTAT‐3′) and DQAdown (5′‐AAGAGGCAGAGCATTGGACA‐3′; Zhu et al., 2007). PCR was performed in a total volume of 30 μl containing 0.75 U of HotStarTaq polymerase (Qiagen, Hilden, Germany), 1× PCR buffer, 60 mM of each dNTP, 10 pM of each primer, 1x BSA (Promega, Madison, USA) and ~10 ng of genomic DNA. Each sample was amplified twice independently at the three loci to ensure positive PCR products. Purified PCR fragments were cloned using the pMD18‐T vector system (Takara, Dalian, China) and then transformed into *Escherichia coli* competent cells (Tiangen, Beijing, China). At least six positive clones per individual were selected at random for Sanger sequencing. Sequencing reactions were performed using an ABI 3730xl DNA Analyzer (Applied Biosystems, Foster City, CA, USA). Because there are two DQA loci, up to four unique sequences at the DQA gene were detected per individual. We selected at least nine positive clones at each DQA locus per individual for sequencing. The identical sequences from at least two clones were defined as an allele. Alleles found only once were verified by additional clone sequencing of the individual. We also designed the primer pairs GP‐DQA‐F (5′‐ATCTGTTTTCTACTTCTTGCTC‐3′) and GP‐DQA‐R (5′‐AGTGAATGAGACCTGGTGTGTA‐3′) for direct sequencing of PCR products to check for agreement with polymorphic sites of the cloned sequences at the DQA loci.

To ascribe the DQA alleles to a specific locus, for the MHC class II DQA1 locus, samples were genotyped using the primer pairs DQA1UP3B (5′‐GTTTAGTAATCATCTTTTCTCCC‐3′) and DQA1DN2 (5′‐ AGAGGCAGAGCATTGGACACATAC‐3′; Chen et al., 2010). We modified two nucleotide bases in the forward primer DQA1UP3B according to the giant panda genome sequence downloaded from Ensembl (http://www.ensembl.org/info/data/ftp/index.html). For the MHC class II DQA2 locus, samples were genotyped using the primer pairs DQA2UP1 (5′‐GTTTCTTCCGTCACTTGGCTTAATAAGG‐3′) and DQA2DN1 (5′‐ AGGCAGAGCATTGGACACATACCAT‐3′; Chen et al., 2010). All amplified alleles were reconstructed using the PHASE function (Stephens & Donnelly, 2003) in DnaSP 5.10 (Librado & Rozas, 2009) with the “recombination” model (−MR0) and 1,000 iterations after 100 burn‐ins (Berggren & Seddon, 2008; Galaverni, Caniglia, Fabbri, Lapalombella, & Randi, 2013). The DQA alleles from the clone sequencing were used as the background for the PHASE analysis. Alleles were matched to sequences available in GenBank via Blastn (Johnson et al., 2008).

When the clone sequencing and PHASE data were combined, most of the individuals from the mating sites and mother‐father‐cub trios were successfully genotyped for the DQA1 and DQA2 loci. Individuals with missing genotypes were excluded in the following data analysis.

### 2.5 Testing the MHC‐based mate choice hypotheses

We tested four mate choice hypotheses based on the MHC data from wild giant pandas, with different estimates of MHC variation for each hypothesis. To determine the MHC‐based heterosis mechanism, we calculated the distribution of homozygotes and heterozygotes at each locus for dominant and subordinate males, respectively. To determine whether MHC genetic diversity affects panda mate choice, two measures were used: (a) the difference in coding amino acids between two haplotypes of an individual (Setchell, Charpentier, Abbott, Wickings, & Knapp, 2010); and (b) the difference in coding amino acids within the antigen‐binding sites (ABS) between two haplotypes of an individual (Setchell et al., 2010). To investigate whether the MHC genetic compatibility hypothesis plays a role in mate choice, three methods were used to calculate the MHC genetic dissimilarity between males and females: (a) the mean number of differences in coding amino acids between female and male alleles using the formula (*P*_CD_ + *P*_Cd_ + *P*_cD_ + *P*_cd_)/4 (Landry, Garant, Duchesne, & Bernatchez, 2001), where *P*_CD_, *P*_Cd_, *P*_cD_, and *P*_cd_ are the number of amino acid differences between two individuals whose genotypes are Cc and Dd; (b) the mean number of differences in coding amino acids within the ABS between female and male alleles, with the same formula as the first method; and (c) twice the number of the proteins female and male individuals share divided by the sum of proteins of both individuals using *D* = 2*Fab*/(*Fa* + *Fb*), where *Fa* and *Fb* are the number of proteins in individuals A and B, and Fab is the number of proteins shared by both individuals (Wetton, Carter, Parkin, & Walters, 1987). At last, to determine whether the MHC‐based “good gene” hypothesis was supported, the allele distribution of each locus between dominant and subordinate males was compared.

### 2.6 Statistical analysis

#### 2.6.1 Correlation test

To detect the impacts of genomic background on MHC variation, we tested the Pearson correlation among female–male relatedness based on microsatellite loci and female–male genetic compatibility parameters based on MHC variation, and tested the correlation among male's genetic heterozygosity based on microsatellite loci and male's MHC genetic diversity parameters, using psych package in RSTUDIO. Based on these correlation test results, we removed one of pairwise significantly correlated parameters for 10 female–male genetic similarity measures and 8 male's genetic diversity measures separately, and retained no significantly correlated parameters for further GLMM analysis.

#### 2.6.2 GLMM analysis

We performed generalized linear mixed model (GLMM) analysis (McCullagh & Nelder, 1989) to assess the roles of MHC heterosis, diversity and compatibility of three MHC class II loci in mating‐pair formation. We also included female–male relatedness and two measures for male's genetic heterozygosity (standardized individual heterozygosity (SH), Coltman, Pilkington, Smith, & Pemberton, 1999; internal relatedness (IR), Amos et al., 2001) based on microsatellite data as the explanatory variables (Hu et al., 2017). The response variable (mating‐pair formation) was defined as binary (“1” denotes the pairing of dominant male and estrous female, and “0” represents the pairing of subordinate male and estrous female), given random effects for different sampling years, mating sites, individual identity or their combinations. The model was fit using the lme4 package in RSTUDIO, with a logit‐link and binomial error distribution.

#### 2.6.3 Chi‐square test

We used the chi‐square test to investigate the good gene hypothesis, and the significance of differences in allele distribution between dominant and subordinate males was tested.

#### 2.6.4 Monte Carlo randomization tests

To detect whether females or males choose partners in a significant, nonrandom way, we compared the partners’ mean MHC genetic dissimilarity with the individuals’ randomized mean obtained by Monte Carlo simulations (Sin et al., 2015). We first calculated the mean MHC genetic dissimilarity for real parent pairs, that is, the mother and father of sampled cubs, then calculated the mean MHC genetic dissimilarity by randomly shuffling all female and male individuals (excluding cubs) 10,000 times. We calculated all sites of coding amino acid sequences and the ABS, respectively.

## 3 RESULTS

### 3.1 Individual identification for mating sites and mother‐cub pairs

From 2008 to 2016, we found 15 mating sites in Foping and four mating sites in Changqing and collected a total of 141 fresh feces and 64 hair samples. Using 14 microsatellite loci, we identified 28 pandas from the Foping mating sites and 12 pandas from the Changqing mating sites, with a gender composition of 26 males and 14 females. Sex identification results confirmed the inference of male and female identity based on reproductive behavior and spatial position clues. Each mating site comprised one estrous female and between 1 and 6 males (see details in Hu et al., 2017).

We found 13 mother‐cub pairs from 2006 to 2012 in Foping and collected 29 feces and 11 hair samples. Mother and cub microsatellite genotypes all conformed to Mendel's law of inheritance. Paternity analysis identified the fathers of 11 panda cubs. Furthermore, for mother‐father‐cub trios, the genotyped MHC alleles at locus DRB1 were consistent with Mendel's law of inheritance, reflecting the reliability of our paternity analysis (see details in Hu et al., 2017).

### 3.2 Genotyping of the DRB1, DQA1, and DQA2 loci

**Figure 2:**
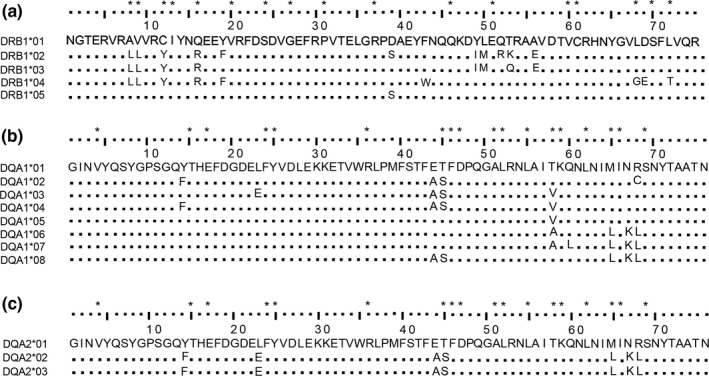
Coding amino acid sequence alignment of the second exon of (a) 5 DRB1 alleles, (b) 8 DQA1 alleles, and (c) 3 DQA2 alleles. Note: “*” above sequences represents putative antigen‐binding sites (ABS) according to Reche and Reinherz (2003). Among them, DQA1*07 and DQA1*08 are two novel alleles found in our study

For the DRB1 locus, we found five alleles in total, all of which had been reported in previous genetic diversity studies (Zhang et al., 2015; Figure 2a). For the DQA1 locus, we found eight alleles in total, including six alleles that had been reported previously (Chen et al., 2013) and two novel alleles that we deposited in GenBank (Aime‐DQA*17‐18, accession numbers: MG050735 and MG050736; Figure 2b). For the DQA2 locus, we found 3 alleles that had been reported previously (Chen et al., 2013; Figure 2c). The corresponding NCBI allele names are listed in Supporting Information Table S1.

### 3.3 Correlation estimate for MHC‐based and microsatellite‐based variables

Several individual samples failed to be amplified successfully due to low quality/quantity of fecal and hair DNA. As a result, for the DQA1 loci, 15 of 19 mating sites were included for the data analysis, while for the DQA2 and DRB1, 16 mating sites were included.

The correlations among male's genetic heterozygosity measures (SH and IR) based on microsatellite loci and male's MHC genetic diversity measures (DQA1_diversity, DQA1_ABS_diversity, DQA2_diversity, DQA2_ABS_diversity, DRB1_diversity, DRB1_ABS_diversity) were estimated (Supporting Information Table S2). Because DQA2 was found to have only three alleles and two kinds of proteins, the very low polymorphism among individuals rendered the correlation measure inapplicable. The results showed that microsatellite‐based SH and IR had significantly high correlation coefficient (*r* = −0.93, *p* < 0.01), while DQA1_diversity and DQA1_ABS_diversity, DRB1_diversity and DRB1_ABS_diversity had significantly high correlation coefficients (*r* = 0.99, *p* < 0.01; and *r* = 0.99, *p* < 0.01), respectively. However, there were no significant correlations between microsatellite‐based genetic heterozygosity and MHC genetic diversity measure (Supporting Information Table S2), suggesting that MHC genetic diversity is not dependent on genome‐scale background diversity.

The correlations among female–male genetic relatedness based on microsatellite loci and female–male genetic compatibility measures based on MHC locus (DQA1_compatibility1, DQA1_ABS_compatibility1, DQA1_compatibility2 DQA2_compatibility, DQA2_ABS_compatibility, DQA2_compatibility2, DRB1_compatibility1, DRB1_ABS_compatibility1, DRB1_compatibility2) were estimated (Supporting Information Table S3). Here, compatibility1 denotes the method from Landry et al. (2001), and compatibility2 denotes the method from Wetton et al. (1987). The results showed that for each of three MHC class II loci, there were significant correlations among compatibility1, ABS_compatibility1, and compatibility2. Among the nine genetic compatibility measures, only DQA1_compatibility2 and DRB1_compatibility2 had significant middle correlation coefficients with female–male relatedness. These results suggest that on the whole, MHC genetic compatibility is not dependent on genome‐scale background relatedness.

### 3.4 Testing mate choice hypotheses using mating site data

Based on the correlation test results, we selected one of significant correlated parameters for further GLMM analysis. As a result, we included as DQA1_heterosis, DQA1_diversity, DQA1_compatibility1, DQA2_compatibility1, DRB1_heterosis, DRB1_diversity, and DRB1_compatibility1 as the explanatory variables, together with microsatellite‐based SH and relatedness. The GLMM analysis (13 mating sites, *n* = 38) showed that neither of these genetic variation measures was significantly associated with mate‐pair formation (Table 1). In addition, to increase sample size and detection power, we performed GLMM analysis for DQA1 (15 mating sites, *n* = 43) and DRB1 loci (16 mating sites, *n* = 43) separately (Supporting Information Tables S4 and S5). However, the two analysis results also detected no significant association between mate‐pair formation and MHC variation measures. These findings suggest that mate‐pair formation could not be explained by MHC heterosis, genetic diversity, and genetic compatibility hypotheses, acknowledging the caveat of the small sample size.

**Table 1:** General linear mixed model (GLMM) of mate‐pair formation, with male's standardized individual heterozygosity (SH), male–female relatedness, DQA1 heterosis, DQA1 diversity, DQA1 compatibility, DQA2 compatibility, DRB1 heterosis, DRB1 diversity, and DRB1 compatibility as explanatory variables, given random effects of year, mating site, and individual identity

	Estimate	Std. Error	*z* Value	Pr(>|*z*|)
(Intercept)	0.15854	2.00481	0.079	0.937
SH	0.09854	2.15564	0.046	0.9635
Relatedness	−3.96687	2.94338	−1.348	0.1777
DQA1_heterosis	2.71903	2.57807	1.055	0.2916
DQA1_diversity	−0.71644	0.54085	−1.325	0.1853
DQA1_compatibility	0.08427	0.55398	0.152	0.8791
DQA2_compatibility^a^	−0.71203	0.97273	−0.732	0.4642
DRB1_heterosis	−1.66383	2.04614	−0.813	0.4161
DRB1_diversity	0.7011	0.39151	1.791	0.0733
DRB1_compatibility	−0.38916	0.2439	−1.595	0.1106

a Due to very low polymorphism in DQA2 locus, the analysis involving DQA2 heterosis and DQA2 diversity was inapplicable.

**Figure 3:**
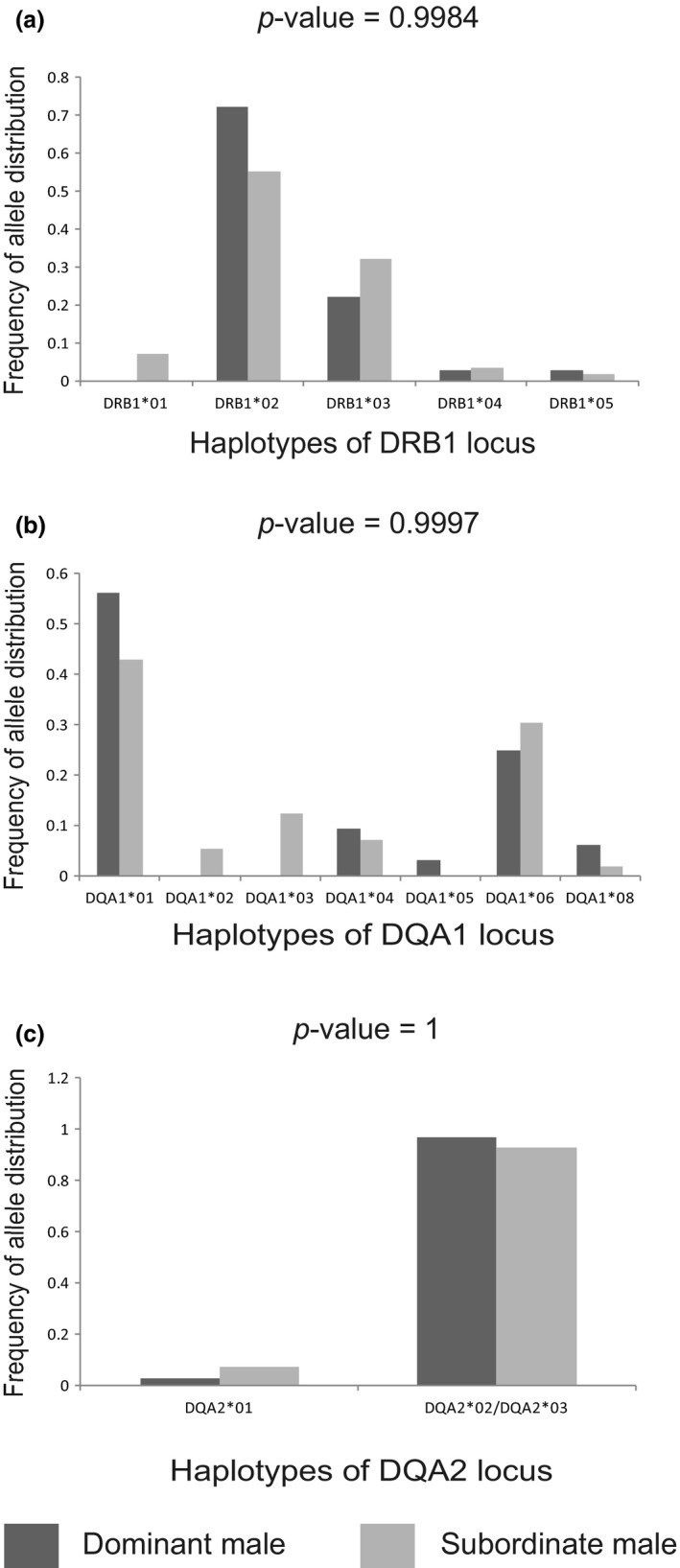
The distribution of alleles at the three MHC loci for testing the good gene hypothesis. (a) the DRB1 locus, (b) the DQA1 locus, and (c) the DQA2 locus. For the DQA2 locus, DQA2*02 and DQA2*03 were combined because of the synonymous nucleotide variation between two genes

Regarding the “good gene” hypothesis, we did not find an MHC allele that had a significant effect on male dominance status. The MHC allele distribution between dominant and subordinate males for the DRB1 (16 mating sites, *n* = 43), DQA1 (15 mating sites, *n* = 43) and DQA2 loci (16 mating sites, *n* = 44) were not significantly different (chi‐square test: for DRB1, *χ* ^2^ = 0.115, *p* = 0.9984; for DQA1, *χ* ^2^ = 0.2609, *p* = 0.9997; and for DQA2, *χ* ^2^ = 9.415e−32, *p* = 1). The DQA2 locus contained one synonymous mutation (encoding the same amino acid sequence) between the DQA2*02 and DQA2*03 alleles. Thus, we combined the two alleles in our calculation (Figure 3).

### 3.5 Testing mate choice hypothesis using parent‐pair data

Of 11 parental pairs, nine pairs were successfully genotyped for the MHC class II DRB1 locus. Due to the low quality/quantity of fecal or hair DNA and the difficulty in amplifying the DQA1 and DQA2 loci, the majority of parent‐pair samples were not successfully genotyped at the DQA1 and DQA2 loci. Thus, we did not perform parent pair data analysis for the DQA1 and DQA2 loci.

We calculated the MHC genetic compatibility parameters at the DRB1 locus. Using a Monte Carlo randomization test, we found that MHC genetic compatibility measures did not differ significantly between the real parent pairs (*n* = 9) and random female–male pairs (*n* = 40) for the coding amino acid sites (3.9722 vs. 4.125, *p* = 0.89) and for the coding amino acids within the ABS (2.52 vs. 2.8126, *p* = 0.62). Indeed, the sample size was very small, which may affect the reliability of our conclusion.

## 4 DISCUSSION

We implemented a long‐term ecological, behavioral and genetic study of wild giant pandas in the Foping and Changqing Nature Reserves by tracking GPS‐collared giant pandas (Hu et al., 2017; Nie et al., 2012; Wei et al., 2014; Zhang et al., 2014). One aim of the long‐term field project is to explore the genetic mechanisms underlying the sexual selection of wild giant pandas. From 2008 to 2016, we collected behavioral and genetic data from 19 mating sites and 13 mother‐cub pairs. Despite our long‐term attempts to find additional mating sites and mother‐cub pairs, the sample sizes were yet small, which is common in endangered species studies. Based on these mating sites and mother‐cub data, Hu et al. (2017) assessed the inbreeding level and inbreeding avoidance mechanisms of giant pandas and found that female–male relatedness and male genetic heterozygosity did not explain the formation of mating‐pairs or parent‐pairs, based on 14 microsatellite loci. These data suggest that other mechanisms may occur during mate choice. Therefore, we investigated whether MHC genes play a role in the mate choice of giant pandas. In this study, we tested four MHC‐based mate choice hypotheses, including the genetic heterosis, genetic diversity, genetic compatibility and “good gene” hypotheses, using three MHC class II loci: DRB1, DQA1, and DQA2. However, we did not find evidence to support MHC‐based mate choice in wild giant pandas.

Similar to our results, other studies of large mammals have failed to find supporting evidence. A study in wild baboons (*Papio ursinus*) found no evidence of mate choice based on MHC dissimilarity, diversity or rare MHC genotypes. This group suggested that group‐living and sex‐biased dispersal could explain the weakened selection for MHC‐based mate choice (Huchard, Knapp, Wang, Raymond, & Cowlishaw, 2010) Kuduk et al. (2014) also found no evidence of MHC affecting mating success in brown bears, suggesting that other mechanisms exist in shaping MHC polymorphisms.

In our study, the lack of MHC‐based mate choice could have multiple explanations. First, as a rare and vulnerable species, the giant panda's reproductive ecology is characterized by very limited mating opportunities (Nie et al., 2012; Pan et al., 2014; Schaller et al., 1985). An estrous female is fertile for approximately 3 days every mating season. Within one mating site, an estrous female is often detected by 1–6 males, which greatly limits access of males. These circumstances are not ideal for male mate choice. Males must invest considerable time and energy in the pursuit of and competition for females, and the number of available estrous females is small. Considering these constraints, giant pandas may use a “better than nothing” mating strategy, that is, a less optimal mate is better than no mate. As Hu et al. (2017) found, giant pandas also did not use female–male relatedness or male genetic heterozygosity to locate a mate. In a similar manner, a study on snub‐nosed monkeys failed to find evidence supporting MHC‐based mate choice mechanisms. They hypothesized that the females’ choices were restricted within a small breeding site (number = 4–6; Yang et al., 2014).

Second, sex‐biased natal dispersal may weaken the selection for MHC‐based mate choice. Hu et al. (2017) found that panda inbreeding is most likely avoided by female‐biased natal dispersal rather than breeding dispersal or active relatedness‐based mate choice. Female‐biased natal dispersal facilitates the spatial segregation of related relatives and reduces the probability of inbreeding, which may reduce the selection pressure for MHC‐based mate choice in giant pandas.

Third, the lack of evidence for MHC‐based mate choice mechanisms does not rule out the possibility of other mechanisms that influence mate choice. The choice of MHC loci may have different impacts on the results. A study on wild gray mouse lemurs exemplified this phenomenon (Huchard, Baniel, Schliehe‐Diecks, & Kappeler, 2013) and found that genetic dissimilar mechanisms of mate choice occurred at the MHC class II DRB locus, but not at the DQB locus. In this study, we only surveyed three MHC class II loci, and more MHC loci such as class I loci could be considered in future studies. The MHC genes are only one of many genetic candidates that may have correlation with inheritable individual fitness. Other genetic candidates such as major urinary proteins (MUPs) can affect individual chemical signals as well, although this phenomenon has only been documented in mice (Brennan, 2004). It is important that body size may impact mate choice. In the recent past, Martin‐Wintle et al. (2015) found that in captive giant pandas, male body mass significantly affected cub production in both female and male panda reproductive performance experiments and significantly affected copulation success in females. Nie et al. (2012) also found that body size may be the primary factor in male dominance status by visually ranking the body size of males involved in the same mating site. These findings are consistent with our field behavior observations that the dominant males often appeared to be stronger than the subordinate males.

At last, our failure to find evidence of mate choice may have resulted from a small sample size and the low diversity in the selected MHC loci. As discussed in Hu et al. (2017), although we performed long‐term fieldwork, the number of mating sites and mother‐cub pairs were yet small, which may affect our conclusions. In addition, we found 5 DRB1 alleles, 8 DQA1 alleles and 3 DQA2 alleles in total. This low level of MHC diversity is consistent with previous MHC studies of giant pandas, indicating the level of vulnerability of giant pandas and the urgency to facilitate gene flow (Wan et al., 2006; Zhu et al., 2007). However, low MHC genetic diversity may not effectively reveal mate choice mechanisms because it could homogenize the distribution of MHC alleles in different individuals, especially at the DQA2 locus, where homozygotes were the majority of individuals.

In conclusion, acknowledging the caveat of the small sample size often encountered in endangered animal studies, we found no evidence to support MHC‐based mate choice mechanisms at three MHC class II loci. Hu et al. (2017) also did not find relatedness‐based mate choice using the same sample set. These findings suggest that due to limited mating opportunities or female‐biased natal dispersal, giant pandas may mate without considering MHC‐based fitness benefits. Moreover, our results suggest that wild giant pandas have low levels of MHC diversity. Hu et al. (2017) also detected a moderate level of inbreeding in wild giant pandas. These results suggest that in giant pandas adaptive genetic variation is relatively poor, and may be lost due to potential inbreeding risk. Together, our findings provide insight into the genetic status and sexual selection of wild giant pandas and highlight the urgency to improve habitat connectivity to facilitate panda dispersal and gene flow among small fragmented giant panda populations.

## CONFLICT OF INTEREST

None declared.

## AUTHOR CONTRIBUTIONS

FWW and YBH designed the study. YBH and YGN performed the field study and collected the genetic samples. YBH, LJY, and LY conducted the genetic analysis and data analysis. LJY, YBH, and FWW wrote the manuscript.

## DATA ACCESSIBILITY

The two MHC DQA1 gene sequences new identified are deposited in GenBank (accessions numbers MG050735 and MG050736).
