# Supplementary material for: No evidence for MHC‐based mate choice in wild giant pandas
Source: Ecol Evol. 2018 Aug 1;8(17):8642–51. doi: 10.1002/ece3.4419 (PMC6157678; doi:10.1002/ece3.4419)
Supplement: Supplementary file 1 [file ECE3-8-8642-s001.docx]

**Supporting information**

**Additional methods**

To better represent the test results of each mate choice hypothesis, we also demonstrated the detailed distribution of MHC variation measures between dominant and subordinate males, and performed single statistical test for each hypothesis.

*Binomial distribution*

We used the binomial distribution to determine whether a male’s dominance status was correlated with heterosis. The frequency of homozygotes and heterozygotes were calculated in dominant and subordinate males.

*Independent sample t test*

We used the independent sample *t* test to determine whether a male’s dominance status was determined by the hypotheses of genetic diversity and genetic compatibility. The significance of differences in the distributions of the measurements of each hypothesis between dominant and subordinate males was tested.

**Fig. S1**

The distribution of heterozygotes and homozygotes at the three MHC loci for testing the genetic heterosis hypothesis. (a) at the DRB1 locus (16 mating sites, n=43), (b) at the DQA1 locus (15 mating sites, n=43), and (c) at the DQA2 locus (16 mating sites, n=44). We found that the distribution of homozygotes and heterozygotes at all three MHC class II loci was not significantly different between dominant males and subordinate males (binomial test).

**
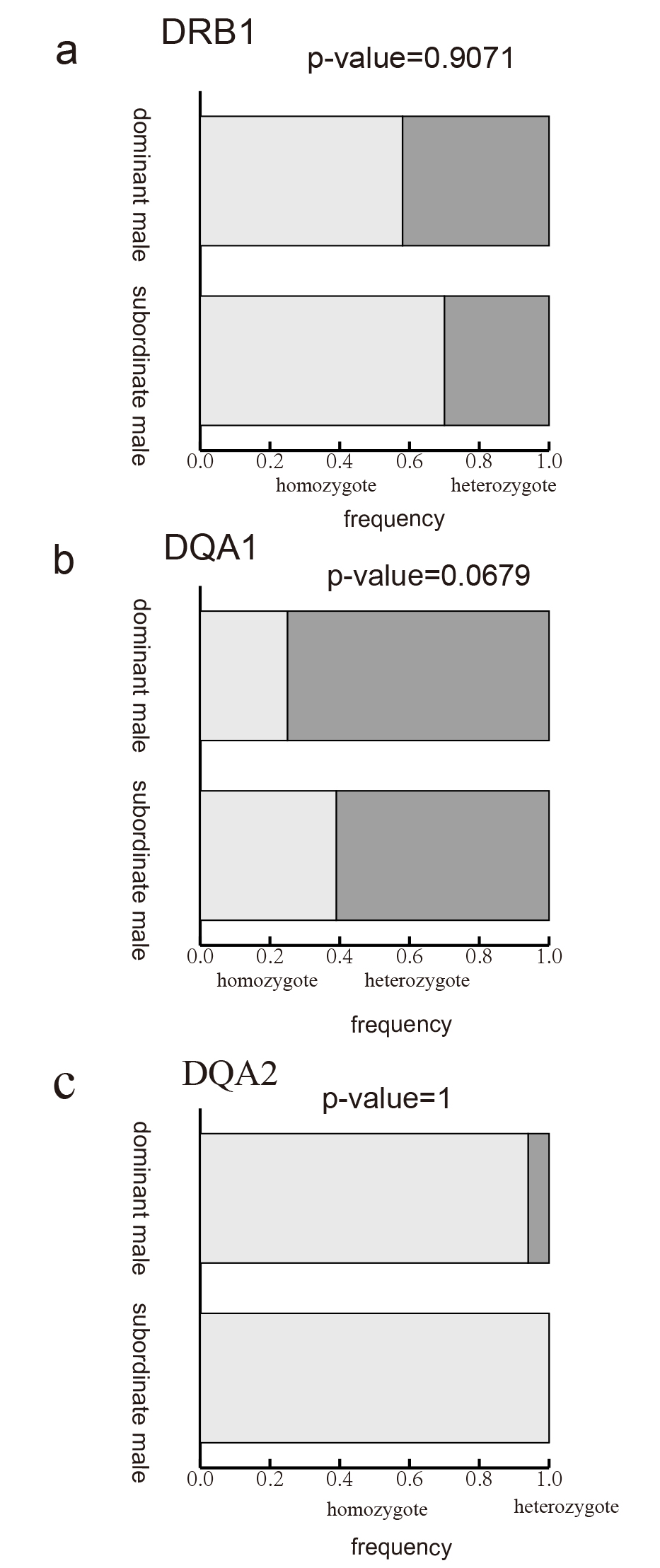
**

**Fig. S2**

The distribution of the measure “genetic diversity” at the three MHC loci. (a) at all sites of the DRB1 locus (16 mating sites, n=43), (b) at ABS of the DRB1 locus, (c) at all sites of the DQA1 locus (15 mating sites, n=43), (d) at ABS of the DQA1 locus, (e) at all sites of the DQA2 locus (16 mating sites, n=44), and (f) at ABS of the DQA2 locus. P values did not differ significantly between dominant males and subordinate males (t test).


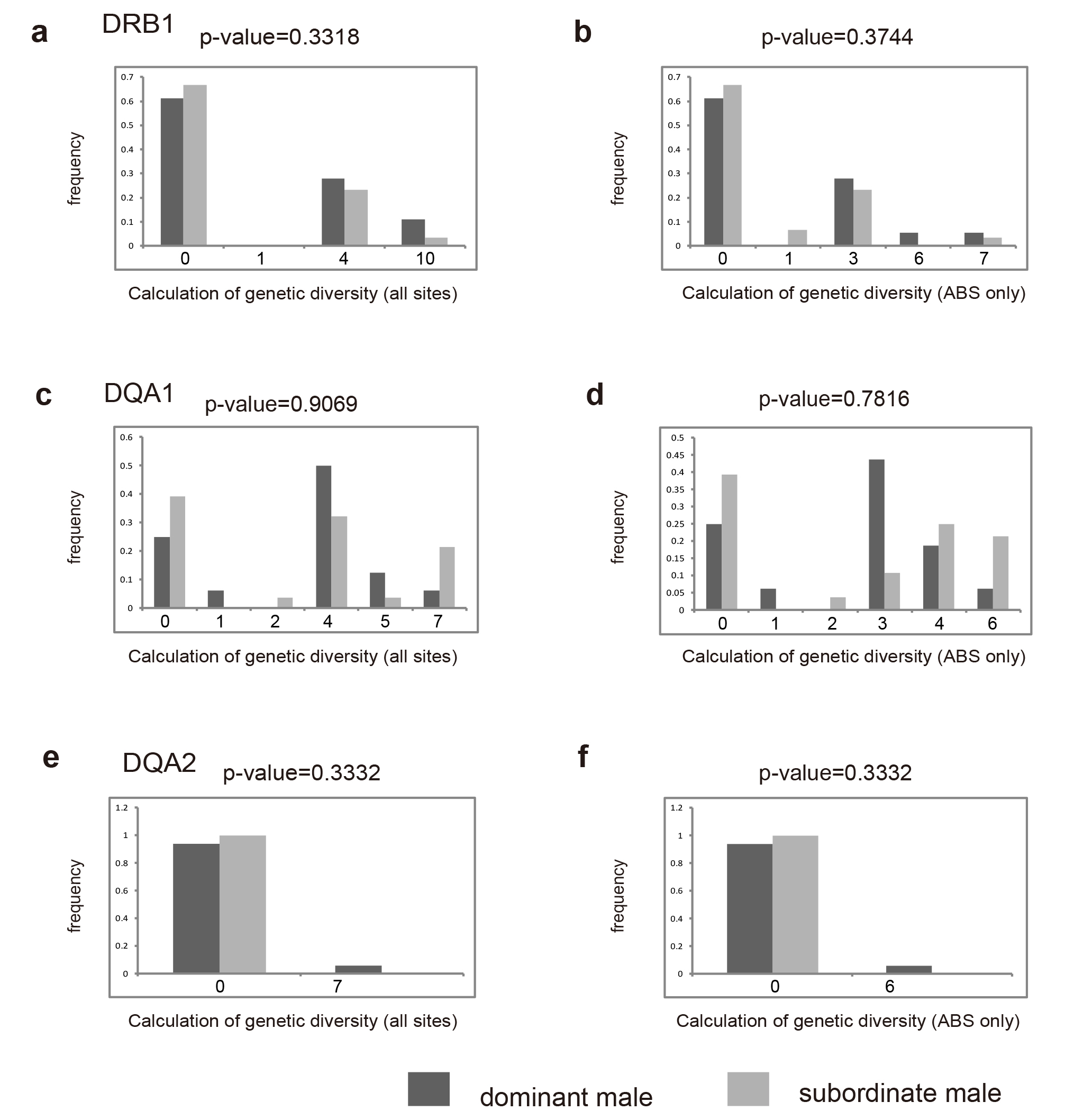


**Fig. S3**

The distribution of the measure “genetic compatibility1” (Landry et al., 2001) at the three MHC loci. (a) at all sites of the DRB1 locus (16 mating sites, n=43), (b) at ABS of the DRB1 locus, (c) at all sites of the DQA1 locus (15 mating sites, n=43), (d) at ABS of the DQA1 locus, (e) at all sites of the DQA2 locus (16 mating sites, n=44), and (f) at ABS of the DQA2 locus. The mean number of differences in coding amino acids between female-dominant male pairs and female-subordinate male pairs did not differ significantly (t test).

**
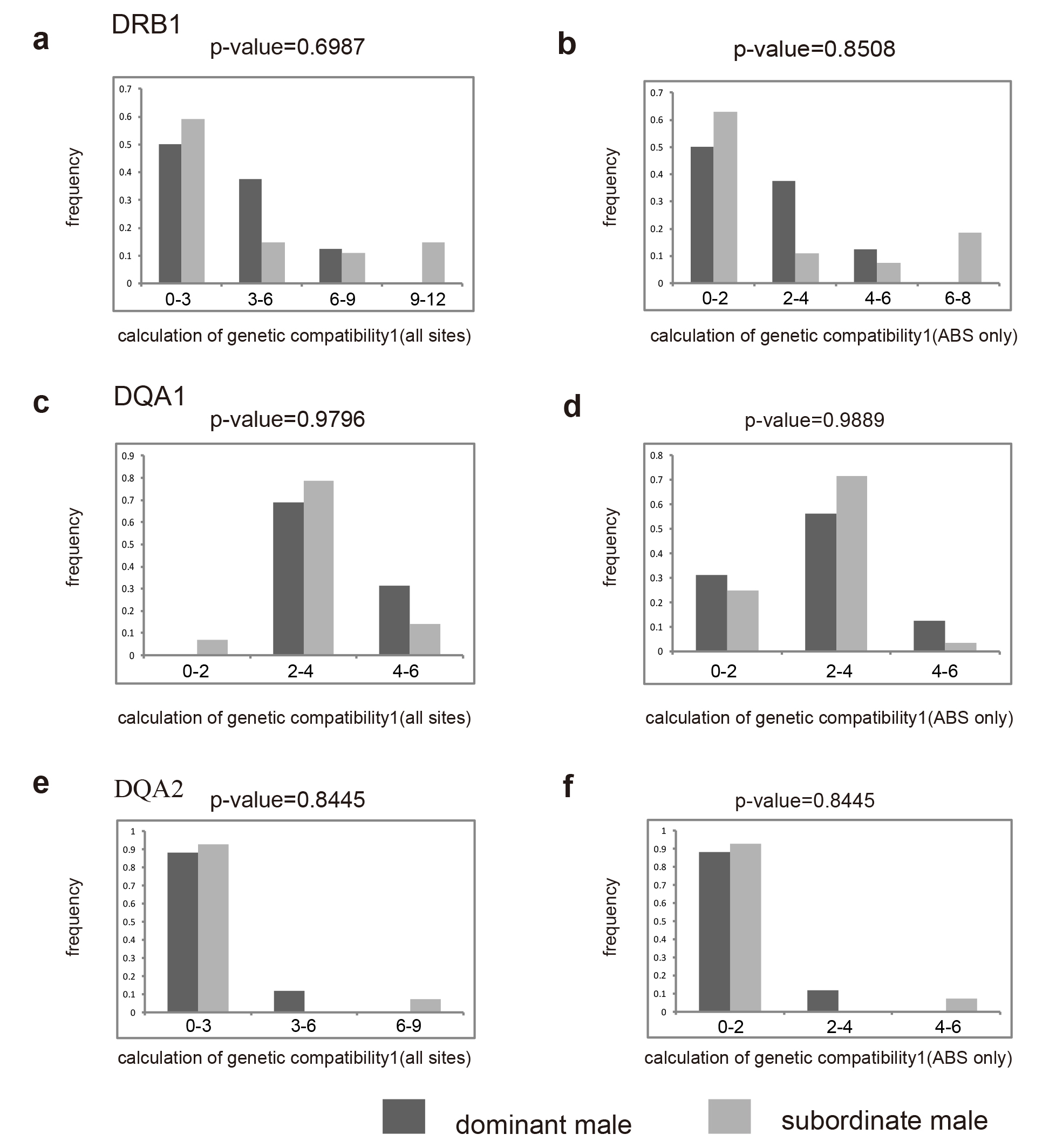
**

**Fig. S4**

The distribution of the measure “genetic compatibility2” (Wetton et al., 1987) at the three MHC loci. (a) at the DRB1 locus (16 mating sites, n=43), (b) at the DQA1 locus (15 mating sites, n=43), (c) at the DQA2 locus (16 mating sites, n=44). The measure did not differ significantly between female-dominant male pairs and female-subordinate male pairs (t test).

**
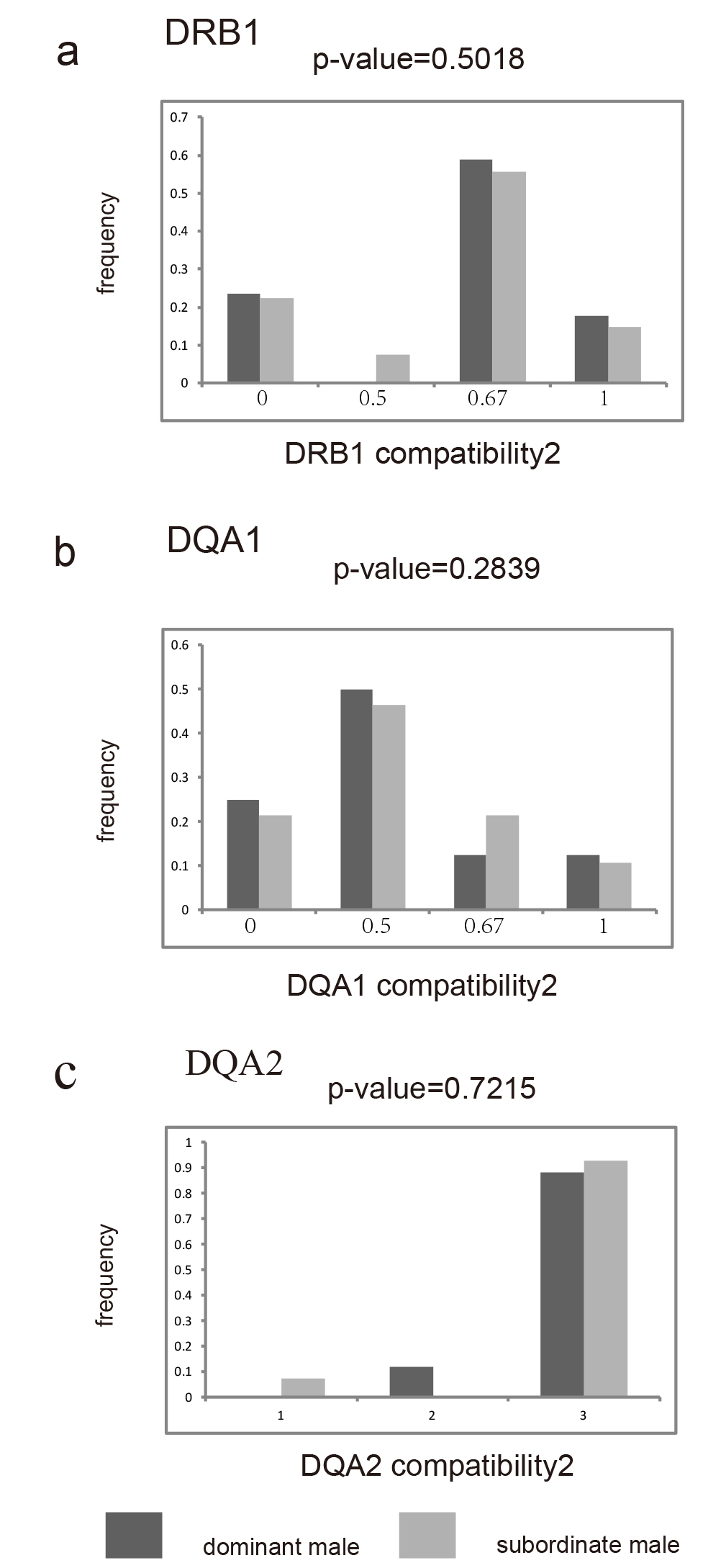
**

**Table S1**

MHC alleles found in our study and corresponding name in the previous studies (Chen et al., 2013; Zhang et al., 2015)

| **DRB1 locus** | **NCBI name** | **DQA1 locus** | **NCBI name** | **DQA2 locus** | **NCBI name** |
| --- | --- | --- | --- | --- | --- |
| DRB1*01 | Aime-DRB*12 | DQA1*01 | Aime-DQA1*03 | DQA2*01 | Aime-DQA2*06 |
| DRB1*02 | Aime-DRB*9 | DQA1*02 | Aime-DQA1*07 | DQA2*02 | Aime-DQA2*01 |
| DRB1*03 | Aime-DRB*8 | DQA1*03 | Aime-DQA1*06 | DQA2*03 | Aime-DQA2*03 |
| DRB1*04 | Aime-DRB*13 | DQA1*04 | Aime-DQA1*02 |  |  |
| DRB1*05 | Aime-DRB*10 | DQA1*05 | Aime-DQA1*01 |  |  |
|  |  | DQA1*06 | Aime-DQA1*05 |  |  |
|  |  | **DQA1*07** |  |  |  |
|  |  | **DQA1*08** |  |  |  |

Bold letter: new alleles found in our study.

**Table S2**

The Pearson correlation between female-male relatedness based on microsatellite loci and female-male genetic compatibility parameters based on MHC variation

|  | Relatedness | DQA1_  compatibility1 | DQA1_ABS_compatibility1 | DQA1_  compatibility2 | DQA2_  compatibility1 | DQA2_ABS_compatibility1 | DQA2_  compatibility2 | DRB1_  compatibility1 | DRB1_ABS_  compatibility1 | DRB1_  compatibility2 |
| --- | --- | --- | --- | --- | --- | --- | --- | --- | --- | --- |
| Relatedness |  |  |  |  |  |  |  |  |  |  |
| DQA1_  compatibility1 | -0.02 |  |  |  |  |  |  |  |  |  |
| DQA1_ABS_  compatibility1 | -0.02 | 1* |  |  |  |  |  |  |  |  |
| DQA1_  compatibility2 | 0.43* | -0.61* | -0.61* |  |  |  |  |  |  |  |
| DQA2_  compatibility1 | 0.13 | -0.06 | -0.07 | -0.19 |  |  |  |  |  |  |
| DQA2_ABS_  compatibility1 | 0.13 | -0.06 | -0.07 | -0.19 | 1* |  |  |  |  |  |
| DQA2_  compatibility2 | -0.13 | 0.09 | 0.1 | 0.17 | -0.99* | -0.99* |  |  |  |  |
| DRB1_  compatibility1 | -0.3 | 0.32 | 0.32 | -0.37* | -0.11 | -0.11 | 0.12 |  |  |  |
| DRB1_ABS_  compatibility1 | -0.23 | 0.33 | 0.32 | -0.33 | -0.11 | -0.11 | 0.12 | 0.98* |  |  |
| DRB1_  compatibility2 | 0.43* | -0.4* | -0.4* | 0.65* | 0.22 | 0.22 | -0.22 | -0.76* | -0.74* |  |

*, P < 0.05.

**Table S3**

The Pearson correlation between male’s genetic heterozygosity based on microsatellite loci and male’s MHC genetic diversity parameters

|  | SH | IR | DQA1_  diversity | DQA1_ABS_diversity | DQA2_  diversity | DQA2_ABS_diversity | DRB1_  diversity | DRB1_ABS_diversity |
| --- | --- | --- | --- | --- | --- | --- | --- | --- |
| SH |  |  |  |  |  |  |  |  |
| IR | -0.93* |  |  |  |  |  |  |  |
| DQA1_  diversity | 0.32 | -0.32 |  |  |  |  |  |  |
| DQA1_ABS_diversity | 0.29 | -0.31 | 0.99* |  |  |  |  |  |
| DQA2_  diversity | NA | NA | NA | NA |  |  |  |  |
| DQA2_ABS_diversity | NA | NA | NA | NA | NA |  |  |  |
| DRB1_  diversity | 0.21 | -0.12 | 0.3 | 0.27 | NA | NA |  |  |
| DRB1_ABS  _diversity | 0.28 | -0.19 | 0.33 | 0.3 | NA | NA | 0.99* |  |

*, P < 0.05.

**Table S4**

General linear mixed model (GLMM) of mate-pair formation, with male’s standardized individual heterozygosity (SH), male-female relatedness, DQA1 heterosis, DQA1 diversity and DQA1 compatibility1 as explanatory variables, given random effects of year, mating site and individual identity

|  | **Estimate** | **Std.**  **Error** | **z value** | **Pr(>\|z\|)** |
| --- | --- | --- | --- | --- |
| **(Intercept)** | -0.18449 | 1.63403 | -0.113 | 0.91 |
| **SH** | -0.71419 | 1.45232 | -0.492 | 0.623 |
| **Relatedness** | -0.77882 | 1.71751 | -0.454 | 0.65 |
| **DQA1_heterosis** | 2.35069 | 1.60801 | 1.462 | 0.144 |
| **DQA1_diversity** | -0.32995 | 0.30542 | -1.08 | 0.28 |
| **DQA1_compatibility1** | -0.08384 | 0.34447 | -0.243 | 0.808 |

**Table S5**

General linear mixed model (GLMM) of mate-pair formation, with male’s standardized individual heterozygosity (SH), male-female relatedness, DRB1 heterosis, DRB1 diversity and DRB1 compatibility1 as explanatory variables, given random effects of year, mating site and individual identity

|  | **Estimate** | **Std.**  **Error** | **z value** | **Pr(>\|z\|)** |
| --- | --- | --- | --- | --- |
| **(Intercept)** | -1.31503 | 1.41931 | -0.926 | 0.354 |
| **SH** | 1.21883 | 1.39361 | 0.875 | 0.382 |
| **Relatedness** | -2.13873 | 1.85589 | -1.152 | 0.249 |
| **DRB1_heterosis** | -1.73734 | 1.33699 | -1.299 | 0.194 |
| **DRB1_diversity** | 0.37487 | 0.23521 | 1.594 | 0.111 |
| **DRB1_compatibility1** | -0.08742 | 0.12768 | -0.685 | 0.494 |

**References:**

Chen, Y. Y., Zhu, Y., Wan, Q. H., Lou, J. K., Li, W. J., Ge, Y. F., & Fang, S. G. (2013). Patterns of adaptive and neutral diversity identify the Xiaoxiangling Mountains as a refuge for the giant panda. PLoS One, 8, e70229.

Landry, C., Garant, D., Duchesne, P., & Bernatchez, L. (2001). ‘Good genes as heterozygosity’: the major histocompatibility complex and mate choice in Atlantic salmon (*Salmo salar*). Proceedings of the Royal Society of London B: Biological Sciences, 268, 1279-1285.

Wetton, J. H., Carter, R. E., Parkin, D. T., & Walters, D. (1987). Demographic study of a wild house sparrow population by DNA fingerprinting. Nature, 327, 147-149.

Zhang, L., Wu, Q., Hu, Y., Wu, H., & Wei, F. (2015). Major histocompatibility complex alleles associated with parasite susceptibility in wild giant pandas. Heredity, 114, 85.
